# A novel human NatA N^α^-terminal acetyltransferase complex: hNaa16p-hNaa10p (hNat2-hArd1)

**DOI:** 10.1186/1471-2091-10-15

**Published:** 2009-05-29

**Authors:** Thomas Arnesen, Darina Gromyko, Diane Kagabo, Matthew J Betts, Kristian K Starheim, Jan Erik Varhaug, Dave Anderson, Johan R Lillehaug

**Affiliations:** 1 Department of Molecular Biology, University of Bergen, N-5020 Bergen, Norway; 2 Department of Surgical Sciences, University of Bergen, N-5020 Bergen, Norway; 3 Department of Surgery, Haukeland University Hospital, N-5021 Bergen, Norway; 4 EMBL, Meyerhofstrasse 1, 69117 Heidelberg, Germany; 5 Institute of Molecular Biology, University of Oregon, Eugene, OR 97403-1229, USA; 6 Catalyst Biosciences, South San Francisco CA 94080, USA

## Abstract

**Background:**

Protein acetylation is among the most common protein modifications. The two major types are post-translational N^ε^-lysine acetylation catalyzed by KATs (Lysine acetyltransferases, previously named HATs (histone acetyltransferases) and co-translational N^α^-terminal acetylation catalyzed by NATs (N-terminal acetyltransferases). The major NAT complex in yeast, NatA, is composed of the catalytic subunit Naa10p (**N a**lpha **a**cetyltransferase **10 p**rotein) (Ard1p) and the auxiliary subunit Naa15p (Nat1p). The NatA complex potentially acetylates Ser-, Ala-, Thr-, Gly-, Val- and Cys- N-termini after Met-cleavage. In humans, the homologues hNaa15p (hNat1) and hNaa10p (hArd1) were demonstrated to form a stable ribosome associated NAT complex acetylating NatA type N-termini *in vitro *and *in vivo*.

**Results:**

We here describe a novel human protein, hNaa16p (hNat2), with 70% sequence identity to hNaa15p (hNat1). The gene encoding hNaa16p originates from an early vertebrate duplication event from the common ancestor of h*NAA15 *and h*NAA16*. Immunoprecipitation coupled to mass spectrometry identified both endogenous hNaa15p and hNaa16p as distinct interaction partners of hNaa10p in HEK293 cells, thus demonstrating the presence of both hNaa15p-hNaa10p and hNaa16p-hNaa10p complexes. The hNaa16p-hNaa10p complex acetylates NatA type N-termini *in vitro*. hNaa16p is ribosome associated, supporting its potential role in cotranslational N^α^-terminal acetylation. h*NAA16 *is expressed in a variety of human cell lines, but is generally less abundant as compared to h*NAA15*. Specific knockdown of h*NAA16 *induces cell death, suggesting an essential role for hNaa16p in human cells.

**Conclusion:**

At least two distinct NatA protein N^α^-terminal acetyltransferases coexist in human cells potentially creating a more complex and flexible system for N^α^-terminal acetylation as compared to lower eukaryotes.

## Background

About 80% of all mammalian proteins and 50% of yeast proteins are estimated to be cotranslationally acetylated at their N-termini [1-6]. This clearly makes N-terminal acetylation one of the most common protein modifications in eukaryotic cells. In yeast, three complexes, NatA, NatB and NatC, express different substrate specificities and are responsible for the majority of N-terminal acetylation [6]. At present, the nomenclature of this class of enzymes is not coherent and later this year a revised nomenclature of this enzyme class will be presented (Polevoda B, Arnesen T and Sherman F, unpublished). In brief, for the proteins mentioned in this study the following names will apply: Naa10p (Ard1), Naa11p (Ard2), Naa15p (Nat1), Naa16p (Nat2) and Naa50p (Nat5). The yeast NatA complex contains the structural subunit Naa15p mediating ribosome association and the catalytic subunit Naa10p [7,8]. Deletion of y*NAA15 *and y*NAA10 *results in a number of common defects including lack of G_o _entry, reduced cell growth, and inability to sporulate [9-11]. The subunit Naa50p is also physically associated with Naa10p and Naa15p, but the function of hNaa50p is unknown [7]. The human NatA, NatB and NatC complexes were recently characterized [12-15]. The human NatA complex contains the human homologues of the yeast NatA components hNaa10p, hNaa15p and hNaa50p [13,16]. The function and substrate specificity of hNatA *in vivo *and *in vitro *were found to resemble that of the yeast NatA complex [1]. The yeast *NAA10 *gene is duplicated in mammals. In humans, the *NAA10 *duplication has lead to the generation of a novel protein designated hNaa11p [17]. Similarly to hNaa10p, hNaa11p potentially interacts with hNaa15p implying that two distinct NatA complexes may exist in human cells: both hNaa15p-hNaa10p and hNaa15p-hNaa11p [17]. However, an endogenous hNaa15p-hNaa11p complex has not yet been detected, thus the functional importance of hNaa11p remains to be elucidated. hNaa10p and hNaa15p were previously demonstrated to be important for normal cellular viability. RNA interference-mediated knockdown of h*NAA10 *or h*NAA15 *induced apoptosis and cell cycle arrest in human cell lines [18-20], thus hNaa10p has been proposed to be a novel cancer drug target [21]. On the other hand, it has also been reported that hNaa10p is essential for the induction of apoptosis since knockdown of hNaa10p protected cells against doxorubicin induced apoptosis [22].

In order to identify novel interaction partners of hNaa10p, we performed immunoprecipitation of hNaa10p from HEK293 cells, followed by trypsin digestion of immunoprecipitated proteins and peptide analysis by mass spectrometry. We here demonstrate the existence of an endogenous hNaa16p protein, encoded by a human paralogue of the h*NAA15 *gene representing a new orthologue of the yeast *NAA15 *gene. h*NAA16 *mRNA is generally expressed in human cells. The hNaa16p protein associates with ribosomes, and interacts with hNaa10p to form a novel human NatA complex.

## Results

### Identification of hNaa16p and the hNaa16p-hNaa10p complex

**Figure 1:**
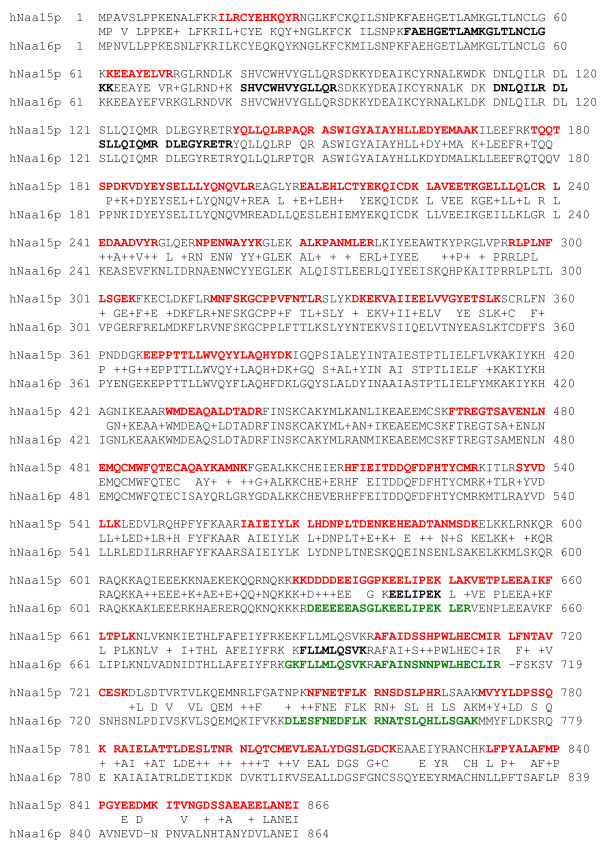
**Alignment of hNaa15p and hNaa16p proteins with identified peptides from anti-hNaa10p affinity extracts indicated**. Sequences for hNaa15p and hNaa16p were aligned using Muscle [36], with sequences common to both proteins shown between the hNaa15p and hNaa16p sequences. Tryptic peptides uniquely identifying hNaa15p are in bold and red, sequences uniquely identifying hNaa16p are in bold and green, and peptides common to both proteins are bold and black. Breaks indicate two different peptides within a region. A number of peptides unique to each protein confirmed that hNaa16p has a different sequence from hNaa15p.

**Figure 2:**
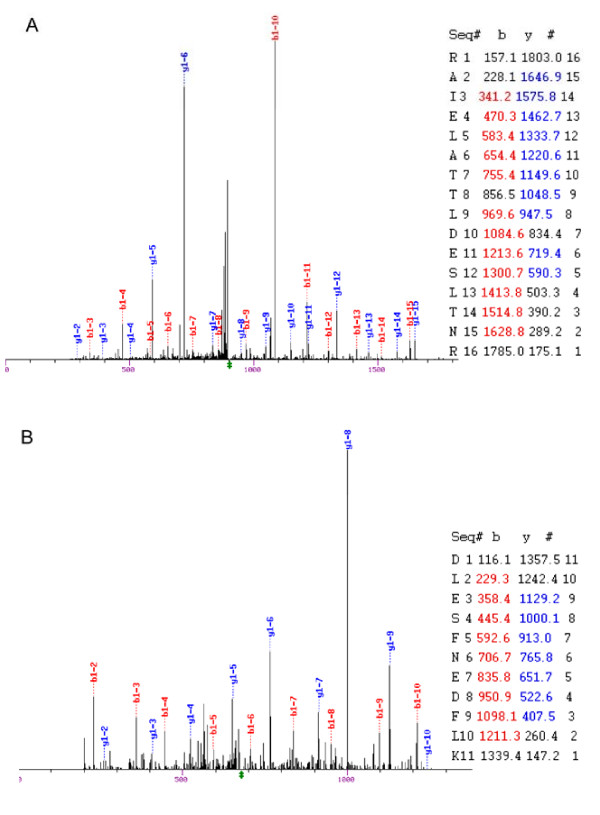
**MS/MS spectra of peptides uniquely identifying hNaa15p and hNaa16p in complex with hNaa10p**. A. MS/MS spectrum of the peptide RAIELATTLDESLTNR, which uniquely identifies hNaa15p in the hNaa10p affinity extraction. The MS/MS spectrum of the +2 peptide ion was matched to this sequence with an Xcorr of 4.25 [30]; the peptide had a probability of correct sequencing of 0.99 [32]. B. MS/MS spectrum of the peptide DLESFNEDFLK, which uniquely identifies hNaa16p in the hNaa10p affinity extraction. The MS/MS spectrum of the +2 peptide ion was matched to this sequence with an Xcorr of 3.4; the peptide had a probability of correct sequencing of 0.95. All fragment ions have a +1 charge. Individual matched y ions (blue) and b ions (red) are indicated in bold; the position of the peptide precursor ion is indicated by the dagger; the x-axis units are m/z and the y-axis represents relative peak intensity normalized to the most intense fragment ion.

**Figure 3:**
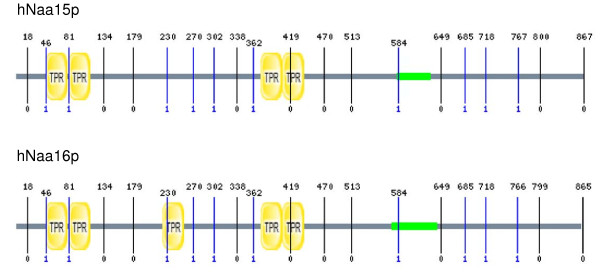
**Overview of structural domains and exon structure of hNaa15p and hNaa16p**. SMART analysis [24] demonstrates the presence of TPR domains (yellow) and coiled coil regions (green). Intron positions are indicated with vertical lines showing the intron phase (below lines) and exact amino acid position (above lines).

We used an immunoprecipitation-mass spectrometry approach to identify novel interaction partners of hNaa10p. Endogenous hNaa10p was collected from HEK293 cell extracts using a hNaa10p-specific antibody. The immunoprecipitates were analysed by LC/MS/MS after trypsin digestion. In addition to hNaa10p, hNaa15p and hNaa50p [13,16], we identified a novel protein, hNaa16p (hNat2/Entrez gene official symbol: NARG1L), in each of four parallel affinity extractions obtained using anti-hNaa10p. Both hNaa15p and hNaa16p were identified by several unique peptides. Neither hNaa10p nor hNaa15p nor hNaa16p were present in four negative controls using unspecific immunoglobulins. The identified hNaa15p and hNaa16p specific peptides are presented in Figure 1 and in Additional File 1. The MS/MS spectra of two of these peptides, RAIELATTLDESLTNR (uniquely identifying hNaa15p) and DLESFNEDFLK (uniquely identifying hNaa16p) are shown in Figure 2. From the alignments (Figure 1 and Additional File 2) it is obvious that the two proteins hNaa15p and hNaa16p are highly similar. According to NCBI bl2seq [23], hNaa15p and hNaa16p share 70% identity and 85% similarity at the amino acid level. In Figure 3, hNaa15p and hNaa16p structural domains as identified by SMART [24] are presented. Both proteins contain several Tetratricopeptide (TPR) domains which are degenerate 34 amino acids repeats containing a helix-loop-helix presumed to be involved in protein-protein interactions. hNaa15p is predicted to contain 4 TPRs while hNaa16p is predicted to have 5 TPRs. This difference results from program threshold values chosen, and since both proteins are highly similar in this region, it is likely that also hNaa15p may have a fifth TPR domain in the same region. Both proteins contain coiled-coil domains (Figure 3). Also the exon-intron organisation for the two genes is highly similar (Figure 3).

**Figure 4:**
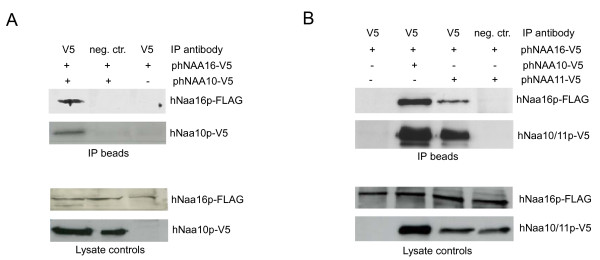
**hNaa10p-V5 and hNaa11p-V5 co-immunoprecipitate hNaa16p-FLAG**. (A) HeLa cells were transfected with ph*NAA10*-V5 and ph*NAA16*-FLAG as indicated and the cell lysates were immunoprecipitated (IP) with anti-V5 or unspecific control antibodies. The immunoprecipitates and lysate controls were analyzed by SDS-PAGE and Western blotting using anti-FLAG and anti-V5 antibodies. The amount of the lysate loaded on the gel represents approximately 20% of the material used for IP. Results are representative of three independent experiments. (B) As (A) using ph*NAA11*-V5 and ph*NAA16*-FLAG plasmids.

The interaction between hNaa16p and hNaa10p appears to be independent of the hNaa15p-hNaa10p complex since hNaa16p-specific peptides were not present in anti-hNaa15p immunoprecipitates analysed in parallel. Since both hNaa15p and hNaa16p interact with hNaa10p, it is of interest to determine the approximate ratio of hNaa15p versus hNaa16p in complex with hNaa10p. To make a rough and qualitative estimate of the amounts of hNaa15p and hNaa16p complexed with hNaa10p, the protein abundance index [25] was calculated for each protein as the number of LC/MS/MS-observed tryptic peptides (unique to each protein) divided by the total number of tryptic peptides that could be identified by SEQUEST under the exact search conditions used. For hNaa15p, this number was 0.0858, while for hNaa16p it was 0.0138. hNaa15p-hNaa10p complexes thus appear to be roughly 6-fold more abundant than hNaa16p-hNaa10p complexes in HEK293 cells. As an additional verification of the hNaa16p-hNaa10p interaction, we expressed tagged hNaa16p-FLAG and hNaa10p-V5 in HeLa cells and demonstrated that hNaa16p-FLAG was co-immunoprecipitated with hNaa10p-V5 (Figure 4A). In conclusion, these observations clearly support the presence of an endogenous hNaa16p-hNaa10p complex in human cells. Furthermore, we also wanted to test whether hNaa16p could interact with hNaa11p, since the latter protein is very similar to hNaa10p. The co-immunoprecipitation experiments using hNaa16p-FLAG and hNaa11p-V5, demonstrated that this indeed is the case (Figure 4B).

### Expression of h*NAA15 *and h*NAA16 *in human cell lines

**Figure 5:**
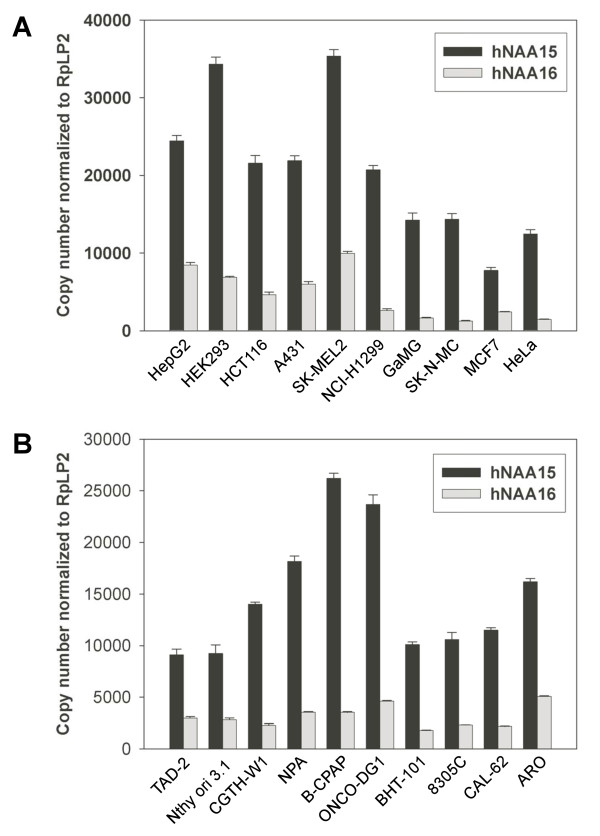
**Expression of h*NAA15 *and h*NAA16 *in human cell lines**. (A) The expression of h*NAA15 *and h*NAA16 *mRNA in 10 different human cell lines was analysed by real-time quantitative PCR (RT-qPCR) using gene specific primers. Values were normalized to the expression of RpLP2. An independent normalization was performed using a second reference gene, RPol2, producing almost identical results (data not shown). The mean ± SD of triplicate experiments was calculated. HepG2, hepatocellular carcinoma; HEK293, kidney adenocarcinoma; HCT116, colon carcinoma; A431, epidermoid carcinoma; SK-MEL-2, malignant melanoma; NCI-H1299, non-small cell lung carcinoma; GaMG, glioblastoma; SK-N-MC, neuroepithelioma; MCF7, breast adenocarcinoma; HeLa, cervix adenocarcinoma. (B) As (A) using 10 different human cell lines originating from the thyroid gland. TAD-2/Nthy ori 3.1, primary human thyroid cells; CGHT-W-1, follicular thyroid carcinoma; NPA/B-CPAP/ONCO-DG-1/BHT-101, papillary thyroid carcinoma; 8305C/CAL-62/ARO, anaplastic thyroid carcinoma.

To investigate the expression of these two human homologues of the yeast *NAA15 *gene, we analysed the expression of h*NAA15 *and h*NAA16 *mRNA by quantitative RT-PCR in 10 human cell lines of various origin (Figure 5A) and 10 human cell lines originating from the thyroid gland (Figure 5B). Both genes are highly expressed in HepG2 (hepatocellular carcinoma), HEK293 (kidney adenocarcinoma), and SK-MEL2 (malignant melanoma) cell lines. Our results demonstrate that h*NAA16 *is expressed in all cell lines analyzed, and h*NAA15 *is the most abundant species of the two. The expression level of h*NAA15 *mRNA is 2–3 times than that of h*NAA16 *mRNA in cell lines like TAD-2 and HepG2, while it is 7–11 times more abundant in GaMG, SK-N-MC, HeLa, and B-CPAP cells. Interestingly, in HEK293 cells, h*NAA15 *mRNA is 5 times more abundant as compared to h*NAA16 *mRNA. Although there is not necessarily a direct relationship between the level of a specific mRNA and the level of the corresponding protein, this agrees with our rough estimates that hNaa15p-hNaa10p complexes appear to be 6-fold more abundant than hNaa16p-hNaa10p complexes in HEK293 cells, thus suggesting that the level of the various NatA complexes present is proportional to the presence of their single subunits. Tissue expression profiles for h*NAA15 *and h*NAA16 *mRNA also indicate that h*NAA15 *mRNA is generally more abundant as compared to h*NAA16 *(NCBI-UNIGENE-EST PROFILE VIEWER). However, in certain tissues like adrenal gland, mammary gland, heart, testis and thymus, h*NAA16 *appears to be the dominant species ( and ). Previously, it was demonstrated that h*NAA15 *was overexpressed in papillary thyroid carcinomas as compared to non-neoplastic thyroid tissue [26,27]. The current data support these observations since h*NAA15 *is slightly or significantly overexpressed in all types of thyroid cancer cell lines tested as compared to the primary thyroid cells (TAD-2/Nthy ori 3.1): follicular thyroid carcinoma (CGTH-W-1), papillary thyroid carcinoma (NPA/B-CPAP/ONCO-DG-1/BHT-101) and anaplastic thyroid carcinoma (8305C/CAL-62/ARO) (Figure 5B). On the other hand, we are not able to make similar conclusions with respect to h*NAA16 *which appears to be expressed equally in tumour- and non-tumour thyroid cell lines.

### Evolution of the h*NAA16 *gene

**Figure 6:**
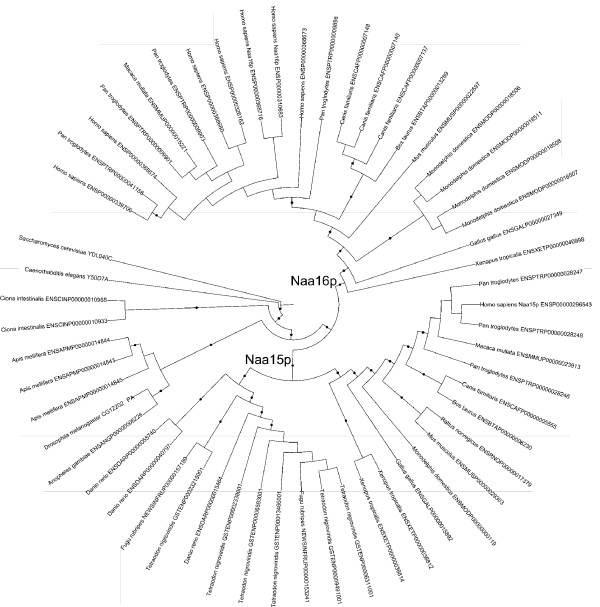
**Phylogenetic tree of *NAA15 *genes**. A phylogenetic tree showing that the duplication leading to h*NAA15 *and h*NAA16 *occurred at some point after the divergence of *Ciona *from the other chordates, and before the speciation that resulted in the amphibia. This is before the duplication resulting in the *NAA10*s [17]. All identifiers are from Ensembl. Branches highlighted with a black dot are those where the probability of the split represented by the branch is > 0.95. Probable *NAA15 *and *NAA16 *clades are indicated. In-paralogues in this tree represent alternative transcripts, which are possibly artefacts of the genome annotation. See Methods for details.

The h*NAA15 *gene (Entrez gene official symbol: *NARG1*) is located to chromosome 4 (4q31.1) while the h*NAA16 *gene (Entrez gene official symbol: *NARG1L*) is located to chromosome 13 (13q14.11). hNaa15p and hNaa16p belong to a protein family with members in *Saccharomyces cerevisiae*, *Caenorhabditis elegans*, *Ciona intestinalis, Drosophila melanogaster*, fish, frog and several higher mammals (Family ENSF00000002142 from Ensembl v38, April 2006 [see methods]). The phylogenetic tree of this family (Figure 6) suggests that the duplication that resulted in h*NAA15 *and h*NAA16 *occurred at some point after the speciation that resulted in the higher chordates (i.e. after the divergence of *Ciona*), but before the speciation of mammals into eutheria and metatheria. The exact timing is confused by *Ciona *branching before *Drosophila*, and by the fish (zebrafish, fugu, tetraodon) appearing in only the *NAA15 *clade (possibly suggesting loss of *NAA16 *in fish). The duplication therefore occurred before the duplication that resulted in h*NAA10 *and h*NAA11*, which we previously narrowed down to some time shortly after the divergence of eutheria and metatheria [17].

Additional File 2 presents an alignment of hNaa15p and hNaa16p protein sequences from human, mouse, and opossum, together with their homologues from *Ciona*, zebrafish and yeast. With the exception of the yeast protein, the sequences are highly similar until approximately 100 residues from their C-termini. The similarity of the zebrafish protein to the Naa15ps rather than the Naa16ps is obvious from the alignment.

### hNaa16p associates with ribosomes and hNaa16p-hNaa10p acetylates NatA-type N-termini *in vitro*

**Figure 7:**
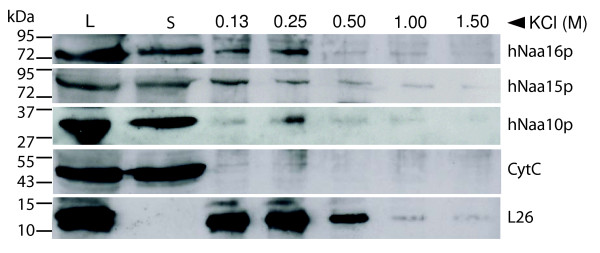
**hNaa16p co-sediments with polysomal fractions in a salt sensitive manner**. Polysomal pellets from HeLa cells expressing hNaa16p-FLAG were resuspended in buffer containing increasing concentrations of KCl. Cell lysate (L), supernatant post first ultracentrifugation (S) and polysomal pellets after KCl treatment were analyzed on SDS-PAGE/Western blotting. The membrane was incubated with anti-FLAG, anti-hNaa15p, anti-hNaa10p, anti-L26 (ribosomal protein) and anti-CytC antibodies. Molecular-mass markers (in kDa) are indicated on the left hand side. Results shown are representative of three independent experiments.

**Figure 8:**
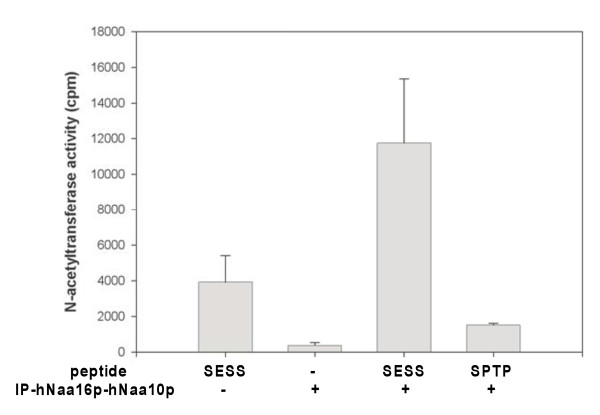
**N-terminal acetyltransferase activity of the hNaa16p-hNaa10p complex**. HeLa cells were transfected with ph*NAA16*-FLAG and ph*NAA10*-V5, and immunoprecipitated with anti-FLAG (+) or a negative control antibody (-). The immunoprecipitated hNaa16p-hNaa10p complex was subjected to an *in vitro *N-acetylation assay by the addition of acetylation buffer, a synthetic oligopeptide, [^14^-C]-Acetyl coenzyme A. The radioactivity incorporated into the peptide was determined by scintillation counting. The peptides used are [H]SESSSKSRWGRPVGRRRRPVRVYP [OH] (SESS) and [H]SPTPPLFRWGRPVGRRRRPVRVYP [OH] (SPTP). An equal level of immunoprecipitated material (input enzyme) was verified by Western-blotting analysis. Results are presented as mean ± SD from three independent experiments.

The characterized hNaa15p-hNaa10p hNatA complex interacts with ribosomes [13] and N-terminally acetylates polypeptides with Ser-, Ala-, Thr-, Val- and Gly- N-termini [1]. Analyses of isolated polysomes demonstrated that hNaa16p-FLAG, as well as hNaa15p and hNaa10p, were both present in the polysomal and the soluble fractions (Figure 7) supporting a model where hNaa16p dynamically associates with ribosomes, and is involved in cotranslational N^α^-terminal acetylation. To assess whether the hNaa16p-hNaa10p complex expresses acetyltransferase activity, we performed *in vitro *N-terminal acetyltransferase assays using immunoprecipitated hNaa16p-hNaa10p and synthetic oligopeptides as substrates. A synthetic oligopeptide representing an acetylated protein N-terminus in HeLa cells, SESS-, was acetylated *in vitro *by hNaa16p-hNaa10p, while an oligopeptide representing an N-terminus not acetylated in HeLa cells, SPTP-, carrying an inhibitory Pro in the second position [1], is not efficiently acetylated *in vitro *(Figure 8). These results suggest that hNaa16p-hNaa10p and hNaa15p-hNaa10p have overlapping substrate specificities, since also the hNaa15p-hNaa10p complex showed a similar *in vitro *reactivity towards these peptides [1]. It should be noted, however, that in order to properly define the substrate specificity of the hNaa16p-hNaa10p complex, a more comprehensive approach is required.

### h*NAA16 *knockdown downregulates hNaa10p and induces cell death

**Figure 9:**
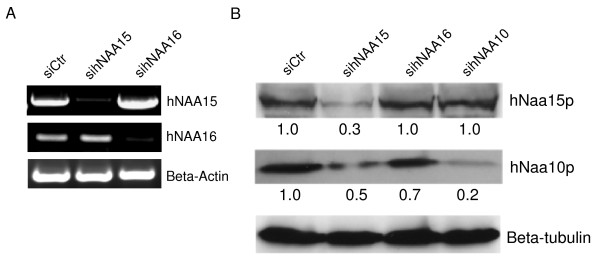
**Knockdown of h*NAA16 *downregulates hNaa10p**. (A) HeLa cells cultured in 6 cm dishes were transfected with 50 nM unspecific (Ctr), h*NAA15 *or h*NAA16 *siRNAs. ZVAD-fmk (5 μM) was added 24 hours post transfection to prevent any induction of apoptosis. After 48 hours total RNA was isolated and processed by RT-PCR with specific primers to h*NAA15*, h*NAA16 *and β-actin. (B) HeLa cells cultured in 6 cm dishes were transfected with 50 nM unspecific (Ctr), h*NAA15*, h*NAA16 *or h*NAA10 *siRNAs. ZVAD-fmk (5 μM) was added 24 and 48 hours post transfection to prevent any induction of apoptosis. After 72 hours cell lysates were analyzed by SDS-PAGE/Western blotting. The membrane was incubated with anti-hNaa10p, anti-hNaa15p and anti-β-tubulin. Results shown are representative of three independent experiments. Protein levels were quantified using FUJIFILM IR LAS 1000 and Image Gauge 3.45. Protein levels in siCtr (-) samples were set to 1.0 and protein levels in sih*NAA15*, sih*NAA16 *or sih*NAA10 *treated cells were estimated relative to this and normalized to β-tubulin levels.

Previously, we demonstrated that siRNA-mediated knockdown of h*NAA10 *or h*NAA15 *induced apoptosis and cell cycle arrest in human cell lines [18]. Using siRNA pools specific for h*NAA15 *and h*NAA16*, we knocked down either gene in HeLa cells. Semiquantitative RT-PCR analysis revealed that both siRNA pools specifically reduced the expression of the targeted gene (Figure 9A). Western blotting analysis of cell lysates harvested 72 hours post siRNA-transfection showed, as expected, that only sih*NAA15*, not sih*NAA16*, reduced the protein levels of hNaa15p (Figure 9B). On the other hand, all three siRNAs sih*NAA15*, sih*NAA16 *and sih*NAA10 *significantly reduced protein levels of hNaa10p. The dependency of hNaa10p for hNaa15p has previously been described [26,28], but the present results also indicate that a fraction of endogenous hNaa10p depend on hNaa16p for stability, albeit to a lesser extent as compared hNaa15p. This is in agreement with the relative presence of hNaa15p-hNaa10p versus hNaa16p-hNaa10p complexes described above.

**Figure 10:**
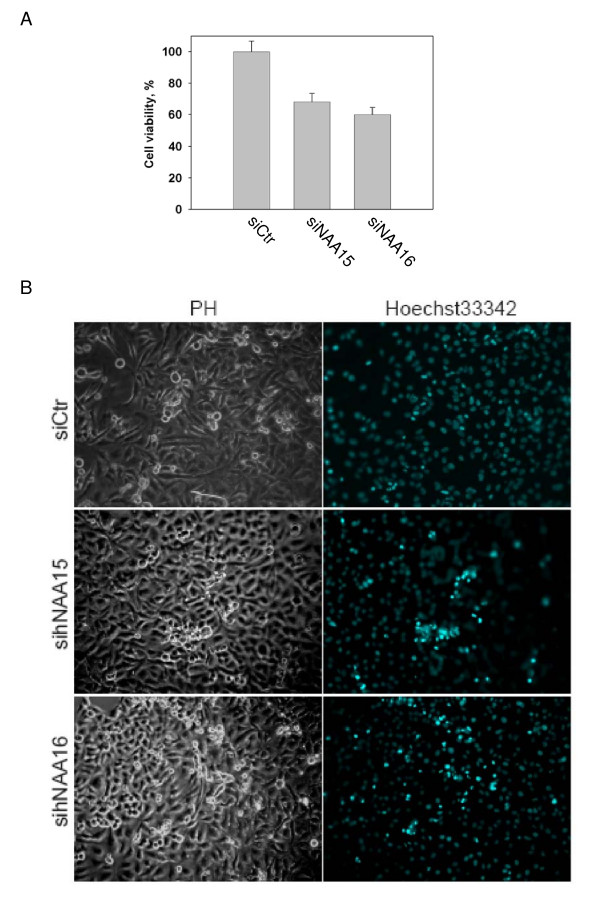
**Knockdown of h*NAA16 *reduces cell viability**. (A) HeLa cells were transfected with 50 nM unspecific, h*NAA15 *or h*NAA16 *siRNAs. Cell viability was determined by WST-1 assay 72 hours post transfection. The viability of the control cells transfected with Ctr siRNA was set to 100%. The mean ± SD of three parallel experiments was calculated. (B) HeLa cells were transfected with 50 nM unspecific, h*NAA15 *or h*NAA16 *siRNAs for 72 hours and analyzed by phase contrast microscopy (PH) to observe cell morphology and Hoechst33342 staining to visualize nuclei.

**Figure 11:**
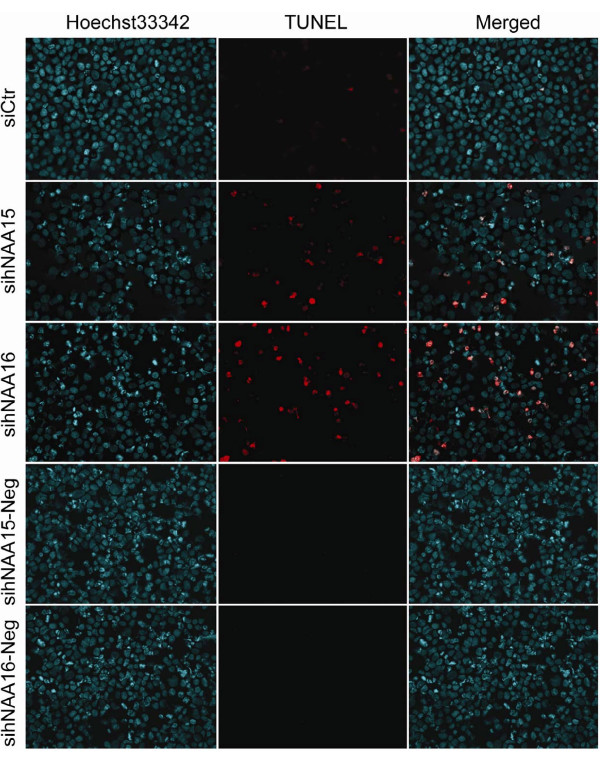
**Knockdown of h*NAA16 *induces cell death**. HeLa cells were transfected with 50 nM unspecific, h*NAA15 *or h*NAA16 *siRNAs. Cells were analyzed by TUNEL-assay 72 hours post transfection. In the left column, Hoechst33342 staining visualizes nuclei in blue; in the middle column, TMR-red TUNEL staining visualizes DNA breaks in red; in the right column, Hoechst and TUNEL signals are merged. Neg: negative controls omitting active TUNEL-reaction enzyme.

Knockdown of h*NAA10 *and h*NAA15 *induces apoptosis in HeLa cells [18], and in the case of these cells, we demonstrated that h*NAA16 *knockdown reduced cell viability at comparable levels to h*NAA15 *knockdown as determined by a WST-1 assay (Figure 10A). In order to investigate this phenotype, we analysed knockdown cells by live microscopy and observed the appearance of dead cells: cells detaching from monolayer had characteristic features of apoptosis (condensation of nucleus, shrinking of cytoplasm, formation of apoptotic bodies). Furthermore, by Hoechst staining we observed a significant increase of pycnotic nuclei in h*NAA15 *and h*NAA16 *knockdown cells as compared to control cells (Figure 10B). A similar observation was made using a TUNEL assay to detect DNA fragmentation further supporting that knockdown of h*NAA16 *induces cell death, most likely apoptosis (Figure 11).

## Discussion

Phylogenetic analysis provides strong support for a vertebrate gene duplication resulting in the two copies of the *NAA15 *gene, possibly followed by loss of the second copy in fish. The presence of hNaa16p specific peptides in four of four parallel anti-hNaa10p immunoprecipitates is a strong indication of the presence of endogenous hNaa16p protein and of the presence of hNaa16p-hNaa10p complexes. Both hNaa15p and hNaa16p are orthologues of the yeast Naa15p. The simultaneous presence of hNaa15p and hNaa16p in anti-hNaa10p immunoprecipitates, and the lack of hNaa16p in anti-hNaa15p immunoprecipitates, indicate that hNaa15p-hNaa10p and hNaa16p-hNaa10p make distinct NatA complexes in the cells. Recently, we also demonstrated the presence of a second human orthologue of the yeast *NAA10 *gene [17]. Thus, two human orthologues of the yeast Naa10p protein exist, hNaa10p and hNaa11p. However, to date there is no evidence of the presence of endogenous complexes consisting of hNaa11p and hNaa15p or hNaa16p. In mouse, two homologues of yeast *NAA15*, m*NAA15 *and m*NAA16 *(named m*NAT1 *and m*NAT2*), were detected at the mRNA level [29]. In this study, m*NAA16 *(mouse homologue of h*NAA16*) was found to be expressed at a lower level as compared to m*NAA15 *(mouse homologue of h*NAA15*), in agreement with our results suggesting that h*NAA15 *is the dominant species as compared to h*NAA16*. Despite the lower abundance of h*NAA16 *mRNA as compared to h*NAA15 *mRNA, we find that h*NAA16 *is generally expressed in a number of human cell lines (Figure 5) and that knockdown of h*NAA16 *induces apoptosis (Figure 11), thus suggesting an important role for this novel gene. Whether the h*NAA16 *knockdown phenotype is resulting from lack of N-terminally acetylated hNaa16p-hNaa10p substrates or whether it is coupled to another unknown function of hNaa16p remains to be investigated.

## Conclusion

In summary, we have identified hNaa16p, a novel hNaa10p interactor. hNaa16p and hNaa10p most likely represent a novel human NatA complex complementing the role of the hNaa15p-hNaa10p complex in protein N^α^-terminal acetylation in human cells. The fact that h*NAA16 *is widely expressed and that h*NAA16 *knockdown induces apoptosis points to hNaa16p as an essential human protein.

## Methods

### Cell culture and transfection

HEK293 cells (embryonal kidney, ATCC CRL-1573) and HeLa cells (human cervix carcinoma, DSMZ no. ACC 57) were cultured at 37°C, 5% CO_2 _in DMEM supplemented with 10% FBS and 3% l-glutamine. Plasmid transfection was performed using Fugene6 (Roche) according to the manufacturers instructions. Plasmid expressing hNaa16p (NARG1L)-FLAG was purchased from Origene (RC214224; NM_024561). Plasmids expressing hNaa10p-V5 and hNaa11-V5 have been described [13,17]. siRNA transfection was performed using Dharmafect (Dharmacon) according to the instruction manual. Gene specific smart pool siRNAs were purchased from Dharmacon and used at a final concentration of 50 nM to silence the h*NAA15 *(h*NAT1/NATH/NARG1) *and h*NAA16 *(h*NAT2*/*NARG1L) *genes: sih*NAA15*, ON-TARGET plus SMARTpool, Cat. L-012847; sih*NAA16*, ON-TARGET plus SMARTpool, Cat. L-013336; negative control (siCtr), ON-TARGETplus Non-Targeting Pool, Cat. no D-001810.

For TUNEL assay, cytospins were prepared and cells were incubated in TUNEL reaction mixture (*In Situ *Cell Death Detection Kit, TMR red, Roche) for 1 hour at 37°C. Negative controls for h*NAA15 *and h*NAA16 *knockdown cells were incubated in TUNEL Label reaction without enzyme.

### Immunoprecipitation and Mass Spectrometry

Immunoprecipitation and Mass Spectrometry were essentially performed as previously described [13], but omitting the crosslinking of antibodies to Protein AG Agarose beads. Rabbit polyclonal peptide specific antibodies against hNaa15p (anti-NATH) and hNaa10p (anti-hARD1) were produced by Biogenes GmBH. The immunogens correspond to amino acids 853–866 of hNaa15p and amino acids 204–217 of hNaa10p. Anti-hNaa10p does not crossreact with hNaa11p since these two proteins are significantly different in the antibody binding region [17]. A Thermofinnigan LCQ Deca XP Plus and an Agilent 1100 Nanoflow hplc were used for microcapillary LC/MS/MS analysis of tryptic peptides. Data analysis utilized SEQUEST [30], Medusa [31] and support vector machine learning [32]. Approximately 5 × 10^7 ^HEK293 cells were used per sample. Four samples were immunoprecipitated in parallel using anti-hNaa15p, four samples using anti-hNaa10p and four samples were immunoprecipitated using rabbit Ig (DAKO) as negative controls. Peptides present in the negative controls were subtracted from the peptides present in the anti-hNaa15p and anti-hNaa10p samples using Medusa [31].

### Immunoprecipitation and Western blotting

Approximately 2 × 10^6 ^cells were harvested for each sample. Cells were lysed in 300 μl IPH lysis buffer (50 mM Tris pH 8, 50 mM NaCl, 0.5% NP-40, 5 mM EDTA, 1 mM Na_3_VO_4_, 1 mM Pefabloc (Roche)) and incubated for 5 minutes on ice. Cell membranes and organelles were removed by centrifugation at 15700 × *g *for 30 seconds, and the cell lysate was transferred to a new tube. To remove proteins that bind unspecifically to the agarose beads, Protein A/G Agarose (Santa Cruz) was added, and the lysate was incubated on a roller for 30 minutes at 4°C. The beads were removed by centrifugation at 1500 × *g *for 4 minutes. The cell lysate was then incubated with 2 μg specific antibody on a roller for 1 – 4 hours at 4°C, before adding 50 μl Prot A/G Agarose beads. The lysate was then incubated on a roller at 4°C for 4 – 10 hours. The beads were collected by centrifuging as above, and washed with 1 × PBS. The supernatant was removed, and the samples were prepared for analysis by SDS-PAGE and Western Blotting as described [21]. Polyclonal rabbit antibodies against hNaa10p and hNaa15p described previously [13], were used at 1:500 dilution, anti-V5 (Invitrogen) at 1:2000, anti-FLAG (Sigma) at 1:2000. Horseradish peroxidase-linked anti-mouse and anti-rabbit were from Amersham Biosciences (Little Chalfont, Bucks., U.K.).

### *In vitro *N^α^-acetyltransferase assay

HEK293 cells were transfected by plasmids as described above and indicated in Figure 8, harvested and lysed in 300 μl IPH lysis buffer. Typically, 5 × 10^6 ^cells were used. 40 μl Protein A/G Agarose (Santa Cruz) was added to the lysates and incubated for one hour at 4°C. After centrifugation at 1500 × *g *for 2 min, the supernatants were collected and incubated for another 2 hours at 4°C with anti-FLAG or unspecific antibody (2 μg). The samples were centrifuged as above and 50 μl Protein A/G Agarose was added to the supernatants. After incubation for 16 hours, centrifugation and three times of washing in 2 × PBS and once in acetylation buffer (50 mM Tris-HCl, pH 8.5, 1 mM DTT, 800 μM EDTA, 10 mM Na-butyrate, 10% Glycerol), the samples were subjected to an *in vitro *acetylation assay. 10 μl peptide (0.5 mM, custom made peptides from Biogenes), 4 μl [^14^C] Acetyl-CoA (50 μCi, 2.07 GBq/mmol, GE Healthcare) and 250 μl acetylation buffer was added to pellets of Protein A/G-Agarose bound hNaa16p-FLAG-hNaa10p-V5 complexes. The mixture was incubated for 2 hours at 37°C with rotation. After centrifugation the supernatant was added to 250 μl SP Sepharose (50% slurry in 0.5 M acetic acid, Sigma) and incubated on a rotor for 5 min. The mixture was centrifuged and the pellet was washed three times with 0.5 M acetic acid and finally with methanol. Radioactivity in the peptide-containing pellet was determined by scintillation counting. All custom made peptides contains 7 unique amino acids at the N-terminus, since these are the major determinants for N-terminal acetylation. The next 17 amino acids are identical to the ACTH peptide (corticotrophin amino acid 1–24) sequence to maintain a positive charge facilitating peptide solubility and effective isolation by cation exchange Sepharose beads. The ACTH derived lysines were replaced by arginines to minimize any potential interference by Nε-acetylation. Peptide sequence information: High mobility group protein A1 (P17096): [H]SESSSKSRWGRPVGRRRRPVRVYP [OH], THO complex subunit 1 (Q96FV9): [H]SPTPPLFRWGRPVGRRRRPVRVYP [OH].

### Isolation of polysomes

Total ribosome isolation was performed as a modification of previously described methods [33,34]. Approximately 2 × 10^7 ^HEK293 cells were used per experiment. Prior to harvesting, cells were treated with 10 μg/ml cycloheximide (CHX) for 5 minutes at 37°C. Cells were harvested, and lysed with KCl ribosome lysis buffer (1.1% (w/v) KCl, 0.15% (w/v) triethanolamine, 0.1% (w/v) magnesium acetate, 8.6% (w/v) sucrose, 0.05% (w/v) Na-Deoxycholate, 0.5% (v/v) Triton-X100, 0.25% (v/v) Pefabloc), and incubated on ice for 15 minutes. After removing nucleus and membranes by centrifugation at 400 × *g *for 10 min, 700 μl cell lysate was ultracentrifuged at 436,000 × *g *for 25 minutes on a 0.4 ml pillow of 25% sucrose in KCl ribosome lysis buffer using a MLA-130 rotor (Beckman, Geneva, Switzerland). Pellets were resuspended in ribosome lysis buffer with the indicated KCl concentrations, followed by ultracentrifugation as described above. Pellets were resuspended in KCl ribosome lysis buffer, and prepared for analysis by SDS-PAGE and Western blot.

### RNA purification and cDNA synthesis

Total RNA was extracted with TRIzol reagent (Invitrogen, San Diego, CA) according to manufacturer's instructions. RNA was subsequently dissolved in DEPC-treated double-distilled water. Single-strand cDNA was synthesized from 1 μg total RNA using Transcriptor Reverse Transcriptase (Roche, Indianapolis, IN) and oligo(dT)_15 _primer according to the manufacturer's instructions.

### Real-time Quantitative PCR

Relative gene expression levels of h*NAA15 *and h*NAA16 *in thyroid cell lines and cell lines from different tissues were determined by real-time quantitative PCR (RT-qPCR).

To amplify h*NAA15 *and *hNAA16 *cDNA the following primers were used: h*NAA15 *primer set 1 (forward TTGGCACGTTTATGGCCTTCT and reverse CGTTTCCCTGTAACCCTCAAGA), h*NAA15 *primer set 2 (forward TGTATGGAGGTATTGGAAGCC and reverse CTCTTCATATCCAGGAGGCAT); h*NAA16 *primer set 1 (forward TCTTCCAGACATTGTGAGCAAAG and reverse AGGTAGCGTTACGTTTCAGAAAA) and h*NAA16 *primer set 2 (forward CAAGATGATTCTGTCGAACCCA and reverse AACGCTGCAAGAGTCCATATAC). For data normalization two reference genes were used independently: large ribosomal protein P2, RpLP2 (GeneID: 6181) and RNA polymerase II, RPol2 (GeneID: 5430). Primers for amplification of reference genes were as following: RpLP2 (forward GACCGGCTCAACAAGGTTAT and reverse CCCCACCAGCAGGTACAC); RPol2 (forward GCACCACGTCCAATGACAT and reverse GTGCGGCTGCTTCCATAA). Templates (equal amount of cDNA) and primers were mixed with components from the LightCycler 480 SYBR Green I Master mix kit (Roche Applied Science). Reactions in triplicate were carried out in the LightCycler 480 real-time PCR machine (Roche Applied Science) under the following conditions: initial denaturation at 95°C for 5 min, and then 40 cycles of denaturation at 95°C for 10 s, annealing at 57°C for 10 s, and extension at 72°C for 10 s. Melting curves were obtained to examine the purity of amplified products. Absolute quantitation data and CP values were obtained by analysis with LightCycler 480 Software 1.5 by 2^nd ^derivative method. By the use of plasmids encoding hNaa15p (ph*NAA15*-V5His) and hNaa16p (ph*NAA16*-Flag), we could verify the specificity of the primers used. The relative amount of h*NAA15*/h*NAA16 *PCR in each sample was normalized to that of RpLP2 PCR and RPol2 independently.

### Alignment and tree building

Homologues to human hNaa15p and hNaa16p were identified using them to search Ensembl version 38, April 2006 [35]. Ensembl peptide identifiers for hNaa15p and hNaa16p are ENSP00000296543 and ENSP000000368716/ENSP00000310683 respectively. The two identifiers for hNaa16p represent two possible alternative transcripts in the Ensembl genome annotation. Both hNaa15p and hNaa16p are members of Ensembl protein family ENSF00000002142. All the peptide sequences from this family were aligned using Muscle [36] with the default settings. Coding sequence alignments were produced by aligning the coding sequences with reference to the alignment of the corresponding peptide sequences. A tree was built from the coding sequence alignment using MrBayes [37] with different rates for transitions and transversions, and running for 250,000 generations. The first 100,000 generations were discarded as burn-in, after which the likelihood scores had converged. A consensus tree was built from the remaining 150,000 generations by sampling every 100th generation, and summarising as a majority-rule consensus tree. The resultant unrooted tree was rooted by treating *Saccharomyces cerevisiae NAA15 *(identifier: YDL040C) as an outgroup. Figure 6 was created with iTOL [38].

## List of abbreviations

ARD1: Arrest defective 1; hNatA: human N-terminal acetyltransferase A; LC: high performance liquid capillary; MS: mass spectrometry; Naa: N alpha acetyltransferase; NAT: N-terminal acetyltransferase.

## Authors' contributions

TA planned the study, performed the large scale immunoprecipitation experiments and wrote the manuscript draft. DG performed knockdown experiments, phenotype studies and quantitative RT-PCR. DK performed immunoprecipitation and acetyltransferase assays. MB performed the evolutionary analysis and alignments. KKS performed polysome experiments. DA performed the mass spectrometry analysis and peptide data analysis. All authors took part in planning and manuscript preparation. All authors read and approved the final manuscript.

## Supplementary Material

Additional File 1 **LC/MS/MS-sequenced peptides identifying hNaa15p and hNaa16p present in quadruplicate hNaa10p affinity extracts**. This table lists all peptides uniquely identifying hNaa15p and hNaa16p and those peptides that are common to both proteins present in hNaa10p affinity extracts. Click here for file

Additional File 2 **Alignment of selected Naa15/16p proteins**. This figure is an alignment of Naa15p and Naa16p protein sequences from human, mouse, and opossum, together with their homologues from *Ciona*, zebrafish and yeast. Click here for file
