# Supplementary material for: A novel human NatA Nα-terminal acetyltransferase complex: hNaa16p-hNaa10p (hNat2-hArd1)
Source: BMC Biochem. 2009 May 29;10:15. doi: 10.1186/1471-2091-10-15 (PMC2695478; doi:10.1186/1471-2091-10-15)
Supplement: Additional File 1 — LC/MS/MS-sequenced peptides identifying hNaa15p and hNaa16p present in quadruplicate hNaa10p affinity extracts. This table lists all peptides uniquely identifying hNaa15p and hNaa16p and those peptides that are common to both proteins present in hNaa10p affinity extracts. [file 1471-2091-10-15-S1.pdf]

ADDITIONAL FILE 1:

LC/MS/MS-sequenced peptides identifying hNaa15p and hNaa16p present in quadruplicate hNaa10p affinity extracts.

**hNaa15p-unique peptides**
AFAIDSSHPWLHEC*MIR
AIELATTLDESLTNR
ALKPANMLER
ASWIGYAIAYHLLEDYEM#AAK
DDDDEEIGGPK
DDDDEEIGGPKEELIPEK
DKEKVAIIEELVVGYETSLK
EALEHLC*TYEK
EALEHLCTYEKQIC*DKLAVEETK
EEPPTTLLWVQYYLAQHYDK
FTREGTSAVENLNEMQC*MWFQTEC*AQAYK
FTREGTSAVENLNEMQCMWFQTECAQAYKAMNK
GC*PPVFNTLR
GELLLQLC*R
HFIEITDDQFDFHTYC*MR
IAIEIYLK
ILRC*YEHKQYR
ITVNGDSSAEAEELANEI
KDDDDEEIGGPKEELIPEK
KEEAYELVR
KKDDDDEEIGGPK
LAKVETPLEEAIK
LAKVETPLEEAIKFLTPLK
LAVEETKGELLLQLC*R
LEDAADVYR
LFNTAVC*ESK
LFPYALAFM#PPGYEEDM#K
LHDNPLTDENKEHEADTANMSDK
LPLNFLSGEK
MNFSKGCPPVFNTLR
MVYYLDPSSQK
MVYYLDPSSQKRAIELATTLDESLTNR
NFNETFLK
NLQTC*M#EVLEALYDGSLGDC*K
NPENWAYYK
RAIELATTLDESLTNR
RLPLNFLSGEK
RNSDSLPHR

SYVDLLK
TQQTSPDKVDYEYSELLLYQNQVLR
VAIIEELVVGYETSLK
VDYEYSELLLYQNQVLR
VETPLEEAIK
WMDEAQALDTADR
YQLLQLRPAQR
**hNaa16p-unique peptides**
AFAINSNNPWLHEC*LIR
DEEEEEASGLKEELIPEK
DEEEEEASGLKEELIPEKLER
DLESFNEDFLK
GKFLLMLQSVK
NATSLQHLLSGAK
RNATSLQHLLSGAK
**peptides common to both**
DLEGYRETR
DLSLLQIQM#R
DNLQILR
EELIPEK
FAEHGETLAM#KGLTLNC*LGKK
FAEHGETLAMK
FLLMLQSVK
GLTLNC*LGK
SHVC*WHVYGLLQR

C* represents cys alkylated with iodoacetamide; M# represents oxidized methionine. Each peptide had a probability of correct sequencing of at least 0.50.
