# Supplementary material for: A novel human NatA Nα-terminal acetyltransferase complex: hNaa16p-hNaa10p (hNat2-hArd1)
Source: BMC Biochem. 2009 May 29;10:15. doi: 10.1186/1471-2091-10-15 (PMC2695478; doi:10.1186/1471-2091-10-15)
Supplement: Additional File 2 — Alignment of selected Naa15/16p proteins. This figure is an alignment of Naa15p and Naa16p protein sequences from human, mouse, and opossum, together with their homologues from Ciona, zebrafish and yeast. [file 1471-2091-10-15-S2.pdf]

```
                1
Human_Naa15p    ---------M PAVS-LPPKE NALFKRILRC YEHKQYRNGL KFCKQILSNP KFAEHGETLA MKGLTLNCLG
Mouse_Naa15p    ---------M PAVS-LPPKE NALFKRILRC YEHKQYRNGL KFCKQILSNP KFAEHGETLA MKGLTLNCLG
Opossum_Naa15p  ---------- ---------- --------RC YEHKQYRNGL KFCKQILSNP KFAEHGETLA MKGLTLNCLG
Zebrafish       ---------M PSIT-LPPKE NALFKRILRC YEHKQYRNGL KFCKQILSNS KFAEHGETLA MKGLTLNCLG
Human_Naa16p    ---------M PNVL-LPPKE SNLFKRILKC YEQKQYKNGL KFCKMILSNP KFAEHGETLA MKGLTLNCLG
Mouse_Naa16p    ---------M PCVQ-LPAKE SALFKRVLKC YEQKQYKNGL KFCKMILSNP KFAEHGETLA MKGLILNCLG
Opossum_Naa16p  ---------M PSVQ-LPPKE SNLFKRILKC YEQKQYKNGL KFCKMILSNP KFAEHGETLA MKGLTLNCLG
Ciona           ---------M PTSQPLPPKE KTLFNRILKC YEQKQYKNGL KFAKQILSNP KFAEHGETLA MKGLTLNCTG
Yeast_Naa15p    MSRKRSTKPK PAAKIALKKE NDQFLEALKL YEGKQYKKSL KLLDAILKKD --GSHVDSLA LKGLDLYSVG

               71
Human_Naa15p    KKEEAYELVR RGLRN---DL KSHVC-WHVY GLLQRSDKKY DEAIKCYRNA LKWDKDNLQI LRDLSLLQIQ
Mouse_Naa15p    KKEEAYELVR RGLRN---DL KSHVC-WHVY GLLQRSDKKY DEAIKCYRNA LKWDKDNLQI LRDLSLLQIQ
Opossum_Naa15p  KKEEAYELVR RGLRN---DL KSHVCSWHVY GLLQRSDKKY DEAIKCYRNA LKWDKDNLQI LRDLSLLQIQ
Zebrafish       RKEEAYDLVR RGLRN---DL KSHVCSWHVY GLLQRSDKKY DEAIKCYRNA LKWDKDNLQI LRDLSLLQIQ
Human_Naa16p    KKEEAYEFVR KGLRN---DV KSHVC-WHVY GLLQRSDKKY DEAIKCYRNA LKLDKDNLQI LRDLSLLQIQ
Mouse_Naa16p    KREEAYEFVR KGLRS---DV RSHVC-WHVY GLLQRSDKKY DEAIKCYRNA LKLDKDNLQI LRDLSLLQIQ
Opossum_Naa16p  KKEEAYEFVR KGLRN---DV KSHVC-WHVY GLLQRSDKKY DEAIKCYRNA LKLDKDNLQI LRDLSLLQIQ
Ciona           KKEEAYELVK RGLKN---DL KSHVC-WHVY GLLQRSDKKY DEAIKCYRNA LRWDKENLHI LRDLSMLQVH
Yeast_Naa15p    EKDDAASYVA NAIRKIEGAS ASPIC-CHVL GIYMRNTKEY KESIKWFTAA LNNGSTNKQI YRDLATLQSQ

              141
Human_Naa15p    MRDLEGYRET RYQLLQLRPA QRASWIGYAI AYHLLEDYEM AAKILEEFRK -TQQTSPDKV DYEYSELLLY
Mouse_Naa15p    MRDLEGYRET RYQLLQLRPA QRASWIGYAI AYHLLEDYEM AAKILEEFRK -TQQTSPDKV DYEYSELLLY
Opossum_Naa15p  MRDLEGYRET RYQLLQLRPA QRASWIGYAI AYHLLEDYEM AAKILEEFRK -TQQTSPDKV DYEYSELLLY
Zebrafish       MRDLEGYRET RYQLLQLRPA QRASWIGYAI AYHLLEDYEM AAKIVEEFRK -TQQTSPDKV DYEYSELLLY
Human_Naa16p    MRDLEGYRET RYQLLQLRPT QRASWIGYAI AYHLLKDYDM ALKLLEEFRQ -TQQVPPNKI DYEYSELILY
Mouse_Naa16p    MRDLEGYRET RYQLLQLRPT QRASWIGYAI AYHLLKDYDT ALKLLEEFRQ -TQQVPPNKI AYEYSELLLY
Opossum_Naa16p  MRDLEGYRET RYQLLQLRPT QRASWIGYAI AYHLLKDYDM ALKLLEEFRK -TQQIPPNKI DYEYSELILY
Ciona           MRDIEGYRET RHALLQLKPG QRAPWIGYAI ANHLLKDYTV AFKVLEEFGK -TQQ----EM VYEHSELLLY
Yeast_Naa15p    IGDFKNALVS RKKYWEAFLG YRANWTSLAV AQDVNGERQQ AINTLSQFEK LAEGKISDSE KYEHSECLMY

              211
Human_Naa15p    QNQVLREA-- ---GLYREAL EHLCTYEKQI CDKLAVEETK GELLLQLCRL EDAADVYRGL QERNPENWAY
Mouse_Naa15p    QNQVLREA-- ---GLYREAL EHLCTYEKQI CDKLAVEETK GELLLQLCRL EDAADVYRGL QERNPENWAY
Opossum_Naa15p  QNQVLREA-- ---GLYREAL EHLGTYEKQI CDKLAVEETK GELLLQLCRL EEATDVYRGL QERNPENWAY
Zebrafish       QNQLLREA-- ---ELFKEAL DHLTSYEKQI CDKLAVEETR GELLLKLDRA GEATEVYHRL LERNPENWSY
Human_Naa16p    QNQVMREA-- ---DLLQESL EHIEMYEKQI CDKLLVEEIK GEILLKLGRL KEASEVFKNL IDRNAENWCY
Mouse_Naa16p    QNQVMREA-- ---NLFQESL EHIETYEKLI CDKLLVEEIK GEMLLKLGRL KEASEVFRNL IDWNAENWCY
Opossum_Naa16p  QNQVMREA-- ---DLFQESL EHIETYEKQI CDKLVVEEIK GEMLLKLGRL KEAGEIFKDL IDRNAENWNY
Ciona           QNYILQEA-- ---GLFQQAI EHLETNKEKI VDKLSWLEYK GDLCVKLERL EEAEKIFTSL VNRNPENHSY
Yeast_Naa15p    KNDIMYKAAS DNQDKLQNVL KHLNDIEPCV FDKFGLLERK ATIYMKLGQL KDASIVYRTL IKRNPDNFKY

              281
Human_Naa15p    YKGLEKALKP ANMLE-RLKI YEEAWTKYPR GLVPRRLPLN FLS-GEKFKE CLDKFLRMNF SKGCPPVFNT
Mouse_Naa15p    YKGLEKALKP ANMLE-RLKI YEEAWTKYPR GLVPRRLPLN FLS-GEKFKE CLDRFLRMNF SKGCPPVFNT
Opossum_Naa15p  YKGLEKSLKP VNMLE-RLKI YEEAWTKYPR GLVPRRLPLN FLSEGEKFKE CLDKFLRMNF SKGCPPVFNT
Zebrafish       YQGLEKALKP KSAED-KQKI YEDAWVKYPK GLVPRRLPLS FLT-GEKFRE CLDRYLRMNF SKGCPPVFTT
Human_Naa16p    YEGLEKALQI STLEE-RLQI YEEISKQHPK AITPRRLPLT LVP-GERFRE LMDKFLRVNF SKGCPPLFTT
Mouse_Naa16p    YEGLEKALQL RSLDE-RLQL YEEVSKQHPR AVSPRRLPLS FAP-GKKFRE LMDKFLRPNF SKGCPPLFTT
Opossum_Naa16p  YEGLEKALQP RTLEE-RLHI YEEISRRHPR AVSPRRLPLN FVP-GEKFRE LMDKFLRVNF SKGCPPLFTT
Ciona           YQKLESVIKP SNEVE-RLQI YDAVSAIFPK ADSPKSLPLT FLT-GPMFQE RVDKIMREQL KRGVPPLFVS
Yeast_Naa15p    YKLLEVSLGI QGDNKLKKAL YGKLEQFYPR CEPPKFIPLT FLQDKEELSK KLREYVLPQL ERGVPATFSN

              351
Human_Naa15p    LRSLY-KDKE KVA-IIEELV VGYETSLKSC RLFNPNDDGK EEPPTTLLWV QYYLAQHYDK IGQPSIALEY
Mouse_Naa15p    LRSLY-RDKE KVA-IVEELV VGYETSLKSC RLFNPNDDGK EEPPTTLLWV QYYLAQHYDK IGQPSIALEY
Opossum_Naa15p  LRSLY-KDKD KVA-IIEELV VGYETSLKSC RLFNPIDDGK EEPPTTLLWV QYYLAQHYDK IGQPSIALEY
Zebrafish       LKSLY-HHKD KVA-IIEELV VGYDKSLKTC KMFNQNDDGK IEPPTTLLWV QYFLAQHFNH IGKHTVALEY
Human_Naa16p    LKSLY-YNTE KVS-IIQELV TNYEASLKTC DFFSPYENGE KEPPTTLLWV QYFLAQHFDK LGQYSLALDY
Mouse_Naa16p    LKSLY-CDTE KVS-IIQELV TNYEASLKMN GYFSPYENGE KEPPTTLLWV QYFLAQHYDK LGQYFLALEY
Opossum_Naa16p  LKSLY-YNTE KVS-MIEELV TNYETSLKTF DYFSPYENGE KEPPTTLLWV RYFLAQHFDK LGQCLLALDY
Ciona           LKPLY-KEQE KVK-CIGEIA EQFIRSLEAN SKFSP-DDEE IEPPTTLLWA RFFYAQHLDK IKEYEKALQL
Yeast_Naa15p    VKPLYQRRKS KVSPLLEKIV LDYLSGLDPT ---------- -QDPIPFIWT NYYLSQHFLF LKDFPKAQEY

              421
Human_Naa15p    INTAIESTPT LIELFLVKAK IYKHAGNIKE AARWMDEAQA LDTADRFINS KCAKYMLKAN LIKEAEEMCS
Mouse_Naa15p    INTAIESTPT LIELFLVKAK IYKHAGNIKE AARWMDEAQA LDTADRFINS KCAKYMLKAN LIKEAEEMCS
Opossum_Naa15p  INAAIESTPT LIELFLVKAK IYKHAGNIKE AARWMDEAQA LDTADRFINS KCAKYMLKAN LIKEAEEMCS
Zebrafish       INTAIESTPT LIELFLIKAK IYKHAGNIRE AARWMDEAQA LDTADRFINS KCAKYLLKAG LIKEAEEMCS
Human_Naa16p    INAAIASTPT LIELFYMKAK IYKHIGNLKE AAKWMDEAQS LDTADRFINS KCAKYMLRAN MIKEAEEMCS
Mouse_Naa16p    INAVIASTPT LIELFYMKAK IYKHMGNLKE AAQWMDEAQS LDTADRFINS KCAKYMLRAN MIKEAEEMCS
Opossum_Naa16p  INAAIASTPT LIELFYIKAK IYKHMGDLKE AAKWMDEAQS LDTADRFINS KCAKYMLRAN MMKEAEEICS
Ciona           INESLEHTPT LIELHVLKAK VLKVRVNMSD ITPSLVGLYA L--IQLWINA KCNIVCLYEQ HLKLNETLSS
Yeast_Naa15p    IDAALDHTPT LVEFYILKAR ILKHLGLMDT AAGILEEGRQ LDLQDRFINC KTVKYFLRAN NIDKAVEVAS
```

```
                491
Human_Naa15p     KFTR--EGTS AVENLNEMQC MWFQTECAQA YKAM------ ---------- ---------- ----------
Mouse_Naa15p     KFTR--EGTS AVENLNEMQC MWFQTECAQA YKAM------ ---------- ---------- ----------
Opossum_Naa15p   KFTR--EGTS AVENLNEMQC MWFQTECAQA YKAM------ ---------- ---------- ----------
Zebrafish        KFTR--EGTS AVENLNEMQC MWFQTECALA YKSL------ ---------- ---------- ----------
Human_Naa16p     KFTR--EGTS AMENLNEMQC MWFQTECISA YQRL------ ---------- ---------- ----------
Mouse_Naa16p     RFTR--EGTS AMENLNEMQC MWFETECISA YQRL------ ---------- ---------- ----------
Opossum_Naa16p   KFTR--EGTS AMENLNEMQC MWFQTECASA YQRL------ ---------- ---------- ----------
Ciona            Q-----ENVS IDEYLKEMQC MWYEVACASA HSRA------ ---------- ---------- ----------
Yeast_Naa15p     LFTKNDDSVN GIKDLHLVEA SWFIVEQAEA YYRLYLDRKK KLDDLASLKK EVESDKSEQI ANDIKENQWL

                561
Human_Naa15p     -NKF-GEALK KCHEIERHFI EITDDQFDFH TYCMRKITLR SYVDLLKLED VLRQHPFYFK AARIAIEIYL
Mouse_Naa15p     -NKF-GEALK KCHEIERHFI EITDDQFDFH TYCMRKITLR SYVDLLKLED VLRQHPFYFK AARIAIEIYL
Opossum_Naa15p   -NKF-GEALK KCHEIERHFV EITDDQFDFH TYCMRKITLR SYVDLLKLED VLRQHPFYFK AARIAIEIYL
Zebrafish        -NKY-GEALK KCHEIERHFV EITDDQFDFH TYCMRKMTLR SYVDLLNLED VLRMHPFYFK AARTAIEIYL
Human_Naa16p     -GRY-GDALK KCHEVERHFF EITDDQFDFH TYCMRKMTLR AYVDLLRLED ILRRHAFYFK AARSAIEIYL
Mouse_Naa16p     -GRY-GDALK KCHEVERHFL EITDDQFDFH TYCMRKMTLR AYVGLLRLED ALRRHTFYFK AARSAIEIYL
Opossum_Naa16p   -GKY-GEALK KCHEIERHFF EITDDQFDFH TYCMRKMTLR AYVDLLRLED ILRKHAFYFK AAQSAVEIYL
Ciona            -NRY-GEALK KCHEVDKHFT EIIEDQFDFH QYCMRKMTLR SYVKMLKLED SLRSHRFYFQ AAKAAIKVYI
Yeast_Naa15p     VRKYKGLALK RFNAIPKFYK QFEDDQLDFH SYCMRKGTPR AYLEMLEWGK ALYTKPMYVR AMKEASKLYF

                631
Human_Naa15p     KLHDNPLTDE NKEHEADTAN MSDKELKKLR NKQRRAQKKA QIEEEKKNAE K----EKQQR NQKKKKDDDD
Mouse_Naa15p     KLHDNPLTDE NKEHEADTAN MSDKELKKLR NKQRRAQKKA QIEEEKKNAE K----EKQQR NQKKKKDDDD
Opossum_Naa15p   KLHDNPLTDE NKEHEADTAN MSDKELKKLR NKQRRAQKKA QHENRKFKWE M----GEQTF NQRYLTSKIC
Zebrafish        SLHDNPLSDD NKESQADNAN LTDKELKKLR NKQRRAQKKA QLEEEKKNAE K----EKQLK NQKKKKEEDE
Human_Naa16p     KLYDNPLTNE SKQQEINSEN LSAKELKKML SKQRRAQKKA KLEEERKHAE R----ERQQK NQKKKRDEEE
Mouse_Naa16p     KLHDNPLTND SKQQDIDSEN LSAKEMKKML SKQRRAQKKA KVEEERKHTE R----ERQQK NQKKKREEEE
Opossum_Naa16p   KLHDNPSTNE RKEQEVNSEN ISAKELKKML SKQRRAQKKA KLEEERKHAE R----ERQQK NQKKKRDEEE
Ciona            HLHDEPYQPG DRIQDEKESS MSNAELKKLR NKQRRQERKK AAAEERKKNE QHDKSGKKKQ QVKNNKTEDD
Yeast_Naa15p     QMHDDRLKRK SDSLDENSDE IQNNG-QNSS SQKKKAKKEA AAMNKRKETE A--------K SVAAYPSDQD

                701
Human_Naa15p     EEIGGPKEEL IPEKLAKVET PLEE-AIKFL TPLKNLVKNK IETHLFAFEI YFRKEKFLLM LQSVKRAFAI
Mouse_Naa15p     EEIGGPKEEL IPEKLAKVET PLEE-AIKFL TPLKNLVKNK IETHLFAFEI YFRKEKFLLM LQSVKRAFAI
Opossum_Naa15p   LKGKLSKASL INSALGGVET PLEE-AIKFL TPLKNLVKNK IETHLFAFEI YFRKEKFLLM LQSVKRAFAI
Zebrafish        EEIGGPKEEL IPEKLAKIEN PLEE-AVKFL TPLKNLVKNK IETHLLAFEI YLRKEKYLLM LQSVKRAYSL
Human_Naa16p     EEASGLKEEL IPEKLERVEN PLEE-AVKFL IPLKNLVADN IDTHLLAFEI YFRKGKFLLM LQSVKRAFAI
Mouse_Naa16p     EVTSGHKEEL IPEKLERVDN PLEE-AIKFL TPLKTLAAES IDTHLLAFEI YFRKGKFLLM LQSVKRAFAI
Opossum_Naa16p   EEASGPKEEL IPEKLEKVEN PLEE-AIKFL IPLKTLVADN IDTHLLAFEI YFRKGKFLLM LQSVKRAFAI
Ciona            AEVKK-EEEF AAEKLVSISN PLEQ-AQKFL APLELYCSGK FETHYLAFQI AERRGTLLVM LRALLRAQKV
Yeast_Naa15p     NDVFG----- --EKLIETST PMEDFATEFY NNYSMQVRED ERDYILDFEF NYRIGKLALC FASLNK-FAK

                771
Human_Naa15p     --DSSHPWLH ECMIRLF-NT AVCESKDLSD TVRTVLKQEM NRLFGATNPK NFNETFLKRN SDSLPHRLSA
Mouse_Naa15p     --DSSHPWLH ECMIRLF-HS -VCESKDLPE TVRTVLKQEM NRLFGATNPK NFNETFLKRN SDSLPHRLSA
Opossum_Naa15p   --DSSHPWLH ECMIRLF-SA AGSESKDLPD AVRTVLKQEM NRLFGATNPK NFNETFLKRN FDSLPHRLSA
Zebrafish        --DPDHPWLH QCLVRFF-KG -VSDSKDMPE AVQTVLKQEI TKLFGESDPK TFNKNFLSKH ANSIPHRVAA
Human_Naa16p     --NSNNPWLH ECLIRFS-KS -VSNHSNLPD IVSKVLSQEM QKIFVKKDLE SFNEDFLKRN ATSLQHLLSG
Mouse_Naa16p     --ESNNPWLH ECLIKFS-KS -VSNHSNLPD IVSKVLAQEM KKIFVNKDLH SFNEDFLRHN ATSLQHLLAG
Opossum_Naa16p   --NSNNPWLH ECLIKFS-RL -VSNHSNLPE IVNKVLTQEM QKIFVNKDLE SFNEEFLKHN ATSIEHLLSG
Ciona            AKESDMPNLH VCRVRFAKKG KFRDAKDLPT AASEVISLHM KIIGCDIDVE TLNEQFIAAN PNSLTHIFAG
Yeast_Naa15p     RFGTTSGLFG SMAIVLL-HA -TRNDTPFDP ILKKVVTKSL EK-------- EYSENFPLNE ISN-------

                841
Human_Naa15p     AKMVYYLDPS SQKRAIELAT TLDESLTNRN LQTCMEVLEA LYDG-SLGDC KEA-AEIYRA NCHKLFPYAL
Mouse_Naa15p     AKMVYYLDSS SQKRAIELAT TLDGSLTNRN LQTCMEVLEA LCDG-SLGDC KEA-AEAYRA SCHKLFPYAL
Opossum_Naa15p   AKMVYYLDPS SQKRAVELAT TLDESLTNRN LQTCMEALGA LCDG-SLGDC KEA-AETYRA NCHKLFPYAL
Zebrafish        AKMMVFLDSS SEGKAAELAT SLNESLTDRS LQTCAEVLQA LRDG-SLGSQ QEKFTESYRV SCHGLYPYAV
Human_Naa16p     AKMMYFLDKS RQEKAIAIAT RLDETIKDKD VKTLIKVSEA LLDG-SFGNC SSQ-YEEYRM ACHNLLPFTS
Mouse_Naa16p     AKMMYFLDKS RQEKAIATAT RLDETIKNKN VKTLIKVSEA LLDG-SFGNC SSQ-YEEYRK TCHSLLPLTP
Opossum_Naa16p   AKMMYFLDES RQEKAIAIAT RLDETVKDKN VKTLTKVFGA LVDG-SFGIC NTQ-YEEYRT ACHKLFPFTS
Ciona            ASSLYHLNSS RKDEALKMIL NINNNMQNVS LKVCEMVLRS LQGKLDFGVV ESDIVAEFRE KCHAIFPRAC
Yeast_Naa15p     ---------- ---NSFDWLN FYQEKFGKND INGLLFLYR- YRDDVPIGSS NLK-----EM IISSLSPLEP

                911
Human_Naa15p     AFMPPGYEED MKITVNGD-- -----SSAEA EELANEI
Mouse_Naa15p     AFMPPGYEED MKITVNGD-- -----SSAET EELANEI
Opossum_Naa15p   AFMPPGYEED TKITINGD-- -----SSAET EELANEI
Zebrafish        AFMPPGYQDN TAVTTNGDL- -----SPCDH EDLPNEM
Human_Naa16p     AFLPAVNEVD NPNVALNH-- ------TANY DVLANEI
Mouse_Naa16p     AFLPAAREAL GLSAELNH-- ------TADH KLLMNEI
Opossum_Naa16p   AFLPSTNEDD SSTISVNQ-- -----TTINH DVMANEI
Ciona            FFLSPQLAAE QNGKVNGHPA VNGLDSNPES GDLKPEH
Yeast_Naa15p     ---------- ---------- ------HSQN EILQYYL
```

Additional File 2 - Alignment of selected Naa15/16p proteins.

An alignment of Naa15p and Naa16p protein sequences from human, mouse, and opossum, together with their homologues from *Ciona*, zebrafish and yeast. Sequence names give the common name of the relevant species and the isoform type where appropriate. These sequences are the same as used in Figure 6: Human_Naa15p = ENSP00000296543, Mouse_Naa15p = ENSMUSP00000029303, Opossum_Naa15p = ENSMODP00000000119, Zebrafish = ENSDARP00000015464, Human_Naa16p = ENSP00000310683, Mouse_Naa16p = ENSMUSP00000022597, Opossum_Naa16p = ENSMODP00000018506, Ciona = ENSCINP00000010933, Yeast_Naa15p = YDL040C.
